# Evaluating construct validity of computable acute respiratory distress syndrome definitions in adults hospitalized with COVID-19: an electronic health records based approach

**DOI:** 10.1186/s12890-023-02560-y

**Published:** 2023-08-09

**Authors:** Neha A. Sathe, Su Xian, F. Linzee Mabrey, David R. Crosslin, Sean D. Mooney, Eric D. Morrell, Kevin Lybarger, Meliha Yetisgen, Gail P. Jarvik, Pavan K. Bhatraju, Mark M. Wurfel

**Affiliations:** 1 Division of Pulmonary, Critical Care and Sleep Medicine, Department of Medicine, University of Washington, 325 9th Avenue HMC #359640, Seattle, WA 98104-2499 USA; 2 Department of Biomedical Informatics and Medical Education, University of Washington, Seattle, WA USA; 3 Division of Biomedical Informatics and Genomics, John W. Deming Department of Medicine, Tulane University School of Medicine, New Orleans, LA USA; 4 Department of Information Sciences and Technology, George Mason University, Fairfax, VA USA; 5 Department of Genome Sciences and Division of Medical Genetics, Department of Medicine, University of Washington Medical Center, Seattle, WA USA

**Keywords:** Electronic health records, Acute respiratory distress syndrome, Phenotyping, Coronavirus disease 2019, Construct validity

## Abstract

**Background:**

Evolving ARDS epidemiology and management during COVID-19 have prompted calls to reexamine the construct validity of Berlin criteria, which have been rarely evaluated in real-world data. We developed a Berlin ARDS definition (EHR-Berlin) computable in electronic health records (EHR) to (1) assess its construct validity, and (2) assess how expanding its criteria affected validity.

**Methods:**

We performed a retrospective cohort study at two tertiary care hospitals with one EHR, among adults hospitalized with COVID-19 February 2020-March 2021. We assessed five candidate definitions for ARDS: the EHR-Berlin definition modeled on Berlin criteria, and four alternatives informed by recent proposals to expand criteria and include patients on high-flow oxygen (EHR-Alternative 1), relax imaging criteria (EHR-Alternatives 2–3), and extend timing windows (EHR-Alternative 4). We evaluated two aspects of construct validity for the EHR-Berlin definition: (1) criterion validity: agreement with manual ARDS classification by experts, available in 175 patients; (2) predictive validity: relationships with hospital mortality, assessed by Pearson *r* and by area under the receiver operating curve (AUROC). We assessed predictive validity and timing of identification of EHR-Berlin definition compared to alternative definitions.

**Results:**

Among 765 patients, mean (SD) age was 57 (18) years and 471 (62%) were male. The EHR-Berlin definition classified 171 (22%) patients as ARDS, which had high agreement with manual classification (kappa 0.85), and was associated with mortality (Pearson *r* = 0.39; AUROC 0.72, 95% CI 0.68, 0.77). In comparison, EHR-Alternative 1 classified 219 (29%) patients as ARDS, maintained similar relationships to mortality (r = 0.40; AUROC 0.74, 95% CI 0.70, 0.79, Delong test P = 0.14), and identified patients earlier in their hospitalization (median 13 vs. 15 h from admission, Wilcoxon signed-rank test P < 0.001). EHR-Alternative 3, which removed imaging criteria, had similar correlation (*r* = 0.41) but better discrimination for mortality (AUROC 0.76, 95% CI 0.72, 0.80; P = 0.036), and identified patients median 2 h (P < 0.001) from admission.

**Conclusions:**

The EHR-Berlin definition can enable ARDS identification with high criterion validity, supporting large-scale study and surveillance. There are opportunities to expand the Berlin criteria that preserve predictive validity and facilitate earlier identification.

**Supplementary Information:**

The online version contains supplementary material available at 10.1186/s12890-023-02560-y.

## Background

Acute respiratory distress syndrome (ARDS) is a common form of hypoxemic respiratory failure with high mortality but few treatments [1, 2]. The high resource utilization and overall burden of the condition was underscored by the coronavirus disease 2019 (COVID-19) pandemic, which has been the most common cause of ARDS and respiratory failure in recent years [3–5]. Scaling ARDS research and surveillance to advance treatment is challenging, because the consensus Berlin definition for the syndrome is complex, subjective, and often demands manual ascertainment. Developing an ARDS definition that is computable in electronic health records (EHR) can enable efficient, reproducible case identification, as research networks and care quality monitoring organizations increasingly use electronically computable definitions to facilitate clinical data collection, track public health case counts, and ensure appropriate care delivery [6–8]. Rapid case identification is especially critical for pandemic preparedness, guiding resource allocation and care decisions [9].

However, the construct validity of the Berlin definition (extent to which the construct captures what it claims to) has been called into question with the evolving epidemiology and treatment of respiratory failure during COVID-19 [10]. There is ongoing discussion about how criteria might be modified to better reflect contemporary management and capture key outcomes [10–12]. To address these gaps our primary aim was to develop a computable ARDS definition consistent with Berlin criteria (EHR-Berlin), and evaluate two indices of construct validity: criterion validity (degree to which the construct compares to accepted standards) and predictive validity (degree to which the construct predicts relevant outcomes) [10, 13, 14]. We hypothesized the EHR-Berlin definition would have high concordance (Cohen’s kappa > 0.80) with classification made by expert clinicians (manual-Berlin), and at least moderate correlations with outcomes (Pearson |*r*| > 0.3, a threshold used for many pulmonary research instruments) [15]. Our secondary aim was to assess how changing timing, oxygenation, and imaging criteria affected the predictive validity of ARDS classification, hypothesizing that expanding criteria can maintain similar relationships to outcomes [14].

## Methods

### Study design, setting and population

**Fig. 1:**
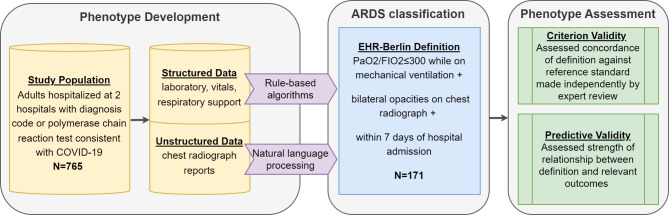
Study Overview. Electronic health records (EHR) data extracted on 765 adults hospitalized with COVID-19 between February 2020 and March 2021. ARDS classifications made by EHR-Berlin definition, which applied rule-based algorithms and natural language processing to EHR data. Our primary aim was to assess two aspects of construct validity for this definition: criterion and predictive validity

An overview of the study design and primary analyses is in Fig. 1. We developed a retrospective cohort of adults hospitalized with COVID-19 at two tertiary care hospitals at University of Washington. From their shared EHR, we extracted data from encounters with a U07.1 International Classification of Diseases Tenth Revision (ICD-10) code or positive polymerase chain reaction consistent with COVID-19 [16].

### Defining EHR-Berlin ARDS

We processed EHR data by applying (1) rule-based algorithms to respiratory support, oxygen saturation, and arterial blood gases with (2) a previously described natural language processing algorithm (NLP) to chest radiograph reports [17]. The NLP algorithm used a neural multitask model to determine whether bilateral opacities were reported; we have previously described high accuracy for this task [17, 18]. The EHR-Berlin definition labeled patients as cases if they met oxygenation criteria (PaO_2_/FIO_2_ ≤ 300 while on invasive or noninvasive mechanical ventilation) within 7 days of hospital admission, and had bilateral opacities on a chest radiograph. We defined our time window from hospitalization, as this often represents a period of worsening respiratory symptoms, and because the exact timing of infection or symptom onset is inconsistently documented in EHR [19]. We used ratio of oxygen saturation to fraction of inspired oxygen (SpO_2_/FIO_2_) ≤ 315 if PaO_2_/FIO_2_ was absent, similar to recent trial protocols adapting to declining use of arterial blood gases [20, 21]. We chose not to incorporate rules for positive end-expiratory pressure because we do not observe levels < 5 cm H_2_O in our system. As this was a cohort of patients hospitalized for COVID-19, we assumed respiratory failure could not fully be explained by cardiac failure or fluid overload, and did not incorporate rules for origin of edema.

### Determining criterion validity of the EHR-Berlin definition

To assess criterion validity, we calculated sensitivity, specificity, positive predictive value (PPV), negative predictive value (NPV), and concordance (Cohen’s kappa) of the EHR-Berlin ARDS definition against manual-Berlin ARDS ascertainment. Manual-Berlin reference labels were determined with chart review by trained research assistants and examination of chest radiographs by a thoracic radiologist or intensivist, and generated independently from EHR-Berlin labels in a subset (n = 175) as part of a published cohort study [22–24].

As exploratory analyses, we evaluated ICD-10 codes and clinician-documented diagnosis against the manual-Berlin reference standard. We were interested in whether these simpler methods, commonly used in administrative and research settings, performed as well our EHR-Berlin definition, which incorporated a range of complex clinical data [25]. Specifically, we examined an ARDS code (J80), alone and combined with acute respiratory failure codes (J96.0, J96.2). Clinician documentation of ARDS was determined by EHR-based text search and manual review of clinical notes.

### Determining predictive validity of the EHR-Berlin definition

We next assessed relationships between the EHR-Berlin definition and key outcomes used in ARDS and COVID-19 trials. Our primary outcome was hospital mortality. We also examined respiratory parameters as secondary outcomes, including ventilator-free days,[26, 27] respiratory support-free days (which included high-flow oxygen, invasive, and noninvasive mechanical ventilation),[28] and WHO ordinal scale ≤ 5 at day 14 [29]. We quantified relationships with Pearson *r* for all outcomes; odds ratios and area under the receiver operating curve (AUROC) for binary outcomes; or beta coefficients for continuous outcomes. Analyses were performed with STATA v17.0.

### Evaluating changes in timing, oxygenation, and imaging criteria of the EHR-Berlin definition

We then sought to clarify how expanding EHR-Berlin criteria would affect predictive validity and prevalence of ARDS classification. We chose *a priori* to focus on the recently proposed modifications:

1. Liberalizing oxygenation criteria by including patients on high-flow oxygen; [11, 12]
2. Liberalizing imaging criteria to include patients with unilateral opacities; [11]
3. Removing imaging criteria for bilateral opacities altogether; [12]
4. Extending timing criteria beyond 7 days [12].

First, we examined these modifications separately. To understand how extending timing criteria could affect case prevalence, we examined the distribution of when patients qualify for the oxygenation and imaging criteria during their hospitalization. Next, we compared outcomes by level of oxygen support (no oxygen, low-flow oxygen by nasal cannula or facemask, high-flow oxygen, or mechanical ventilation) and then by imaging findings (no opacities, unilateral opacities, or bilateral opacities) at admission, in order to understand their standalone predictive validity. We again used univariable logistic and linear regression, with mechanical ventilation and bilateral opacities serving as reference categories, and calculated predicted outcomes in each group with STATA margins function.

Second, we adapted our automated algorithm to develop alternative EHR definitions that applied stepwise the four modifications above (**eTable 1, Supplement**). We assessed predictive validity of alternative definitions with the same methods used to assess the EHR-Berlin definition. Additionally, we evaluated whether these definitions offered better discrimination (with AUROC) for our primary mortality outcome, similar to methods used to optimize case definitions for sepsis and ARDS [30, 31]. Finally, we were interested in whether these definitions identified patients earlier, an oft-cited rationale for expanding Berlin criteria [11]. To achieve this, we focused on patients who eventually met criteria for both Berlin and alternative definitions. We calculated hours between the time patients were admitted to an inpatient service and the time all criteria for each definition were met, and compared this metric with Wilcoxon signed rank test.

## Results

### Baseline clinical features by EHR-Berlin ARDS

We identified 765 adults hospitalized with COVID-19, among which 171 (22%) of patients were classified as EHR-Berlin ARDS (Table 1). These EHR-Berlin ARDS cases were more likely to be male, of Hispanic ethnicity, have diabetes, and have higher baseline illness severity (e.g. need for intensive care unit admission, invasive mechanical ventilation) compared to non-cases.

**Table 1:** Cohort description by EHR-Berlin ARDS phenotype

Features	Total	ARDS-	ARDS+	P
	N = 765	N = 594	N = 171	
**Demographics**				
Age, years, mean (SD)	57 (18)	57 (19)	57 (15)	0.68
Male sex, N (%)	471 (62%)	347 (58%)	124 (73%)	< 0.001
Race, N (%)				0.18
*White*	478 (62%)	372 (63%)	106 (62%)	
*Black/African American*	116 (15%)	100 (17%)	16 (9%)	
*Asian*	99 (13%)	80 (13%)	19 (11%)	
*American Indian or Alaskan Native*	24 (3%)	18 (3%)	6 (4%)	
*Native Hawaiian/ Pacific Islander*	14 (2%)	9 (2%)	5 (3%)	
*Unknown*	34 (4%)	15 (3%)	19 (11%)	
Ethnicity, N (%)				0.016
*Not Hispanic*	539 (70%)	435 (73%)	104 (61%)	
*Hispanic*	176 (23%)	127 (21%)	49 (29%)	
*Unknown*	50 (7%)	32 (5%)	18 (11%)	
**Chronic comorbidities**				
Diabetes, N (%)	314 (41%)	227 (38%)	87 (51%)	0.003
Chronic renal disease, N (%)	202 (26%)	157 (26%)	45 (26%)	0.98
Chronic heart failure, N (%)	199 (26%)	152 (26%)	47 (27%)	0.62
Chronic pulmonary disease, N (%)	174 (23%)	137 (23%)	37 (22%)	0.69
**Illness severity at admission**				
Intensive care unit, N (%)	255 (34%)	121 (20%)	134 (79%)	< 0.001
Invasive mechanical ventilation, N (%)	144 (19%)	32 (5%)	112 (65%)	< 0.001
Noninvasive mechanical ventilation, N (%)	21 (3%)	5 (1%)	16 (9%)	< 0.001
High-flow oxygen, N (%)	42 (5%)	18 (3%)	24 (14%)	< 0.001
**Resource utilization outcomes**				
Duration of invasive mechanical ventilation, days, median (IQR)	0 (0–1)	0 (0–0)	10 (3–22)	< 0.001
ICU length of stay, days, median (IQR)	0 (0–5)	0 (0–1)	13 (4–22)	< 0.001
Hospital length of stay, days, median (IQR)	8 (4–19)	6 (3–13)	19 (10–28)	< 0.001

P values are for two sample t-tests for age, Wilcoxon rank-sum test for resource utilization outcomes, and Chi square tests for categorical variables

#### Criterion validity of the EHR-Berlin definition

There was high agreement between the EHR-Berlin definition and the manual-Berlin reference standard, with kappa = 0.85 (Table 2). Sensitivity was 93% (95% CI 86–97%), specificity was 92% (95% CI 83–97%), and both PPV and NPV exceeded 90%. We performed targeted chart review to better characterize reasons for disagreement (**eTables 2 and 3, Supplement**). Of 7 false negatives (EHR-Berlin negative, manual-Berlin positive), most did not meet imaging criteria with the NLP algorithm. Of 6 false-positives (i.e. EHR-Berlin positive, manual-Berlin negative), most were found to meet all criteria on subsequent chart review, but were not captured initially because the periods of qualifying oxygenation criteria were very brief and missed by manual review.

**Table 2:** Performance of EHR-based strategies to define ARDS compared to manual Berlin reference standard

	EHR-Berlin definition	Diagnosis code (J80)	Diagnosis code combination (J80, J96.0, J96.2)	Clinician-documented diagnosis
Cohen’s kappa	0.85	0.61	0.63	0.70
Sensitivity, %	93 (86–97)	76 (67–84)	80 (81–88)	85 (77–91)
Specificity, %	92 (83–97)	87 (77–93)	84 (73–91)	85 (75–92)
Positive predictive value, %	94 (87–98)	89 (80–94)	87 (79–93)	89 (81–94)
Negative predictive value, %	91 (82–96)	73 (62–82)	76 (65–84)	81 (70–89)

Diagnosis codes from International Classification of Diseases Tenth Revision (ICD-10). Reference standard was ascertained among 175 COVID-19 + patients

In exploratory analyses, we compared simpler EHR-based strategies to identify ARDS, based on diagnosis codes or clinician documentation, to our manual-Berlin reference standard (Table 2). Sensitivity for these ranged from 76% (95% CI 67–84%) for J80 codes to 85% (95% CI 77–91%) for clinician documentation.

### Predictive validity of the EHR-Berlin definition

Next, we examined the strength of relationships between the EHR-Berlin definition and outcomes (Table 3). Compared to non-cases, EHR-Berlin ARDS cases had fewer ventilator-free days and respiratory support-free days; higher mortality; and were less likely to have an ordinal score ≤ 5 at day 14. Correlation between mortality and the EHR-Berlin definition was moderate (*r* = 0.39). The EHR-Berlin definition was more strongly correlated with ventilator-free days (*r* = -0.61), respiratory support-free days (*r* = -0.62), and ordinal score (*r* = -0.59).

**Table 3:** Associations between EHR-Berlin definition and clinical outcomes

	ARDS-	ARDS+	P	*r*	Unadjusted OR or β (95% CI)
	N = 594	N = 171			
Hospital mortality	49 (8%)	73 (43%)	< 0.001	0.39	8.28 (5.43, 12.82)
Respiratory support-free days	28 (27–28)	0 (0–18)	< 0.001	-0.62	-16.67 (-18.16, -15.18)
Ventilator-free days	28 (28–28)	1 (0–21)	< 0.001	-0.61	-16.19 (-17.69, -14.70)
Ordinal score ≤ 5 at day 14	498 (84%)	30 (18%)	< 0.001	-0.59	0.05 (0.03, 0.07)

ARDS- and ARDS + columns indicate N (%) for categorical outcomes (mortality and ordinal score) and median (interquartile range) of continuous outcomes (respiratory support- and ventilator-free days) by patients who were not and who were classified as EHR-Berlin ARDS, respectively. P values are for Chi square tests for categorical outcomes and Wilcoxon rank sum tests for continuous outcomes. *r* indicates Pearson correlation coefficient between EHR-Berlin classification and each outcome. Odds ratio (OR) calculated with logistic regression for categorical outcomes, beta (β) calculated with linear regression for continuous outcomes

### Assessment of timing, oxygenation, and imaging criteria

**Fig. 2:**
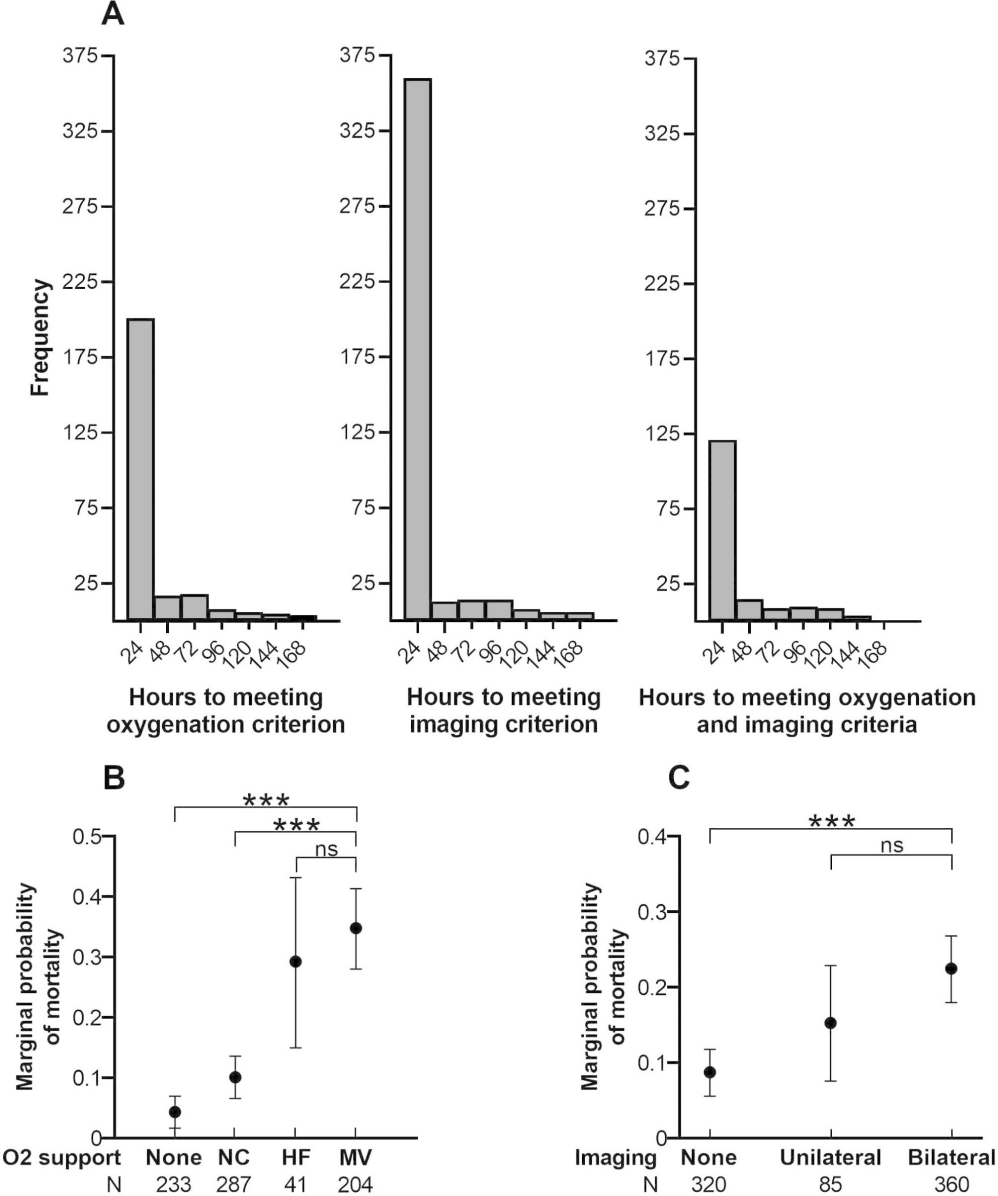
Evaluating timing, oxygenation, and imaging criteria of Berlin definition. Panel **A** shows the frequency distribution of when patients meet oxygenation and imaging criteria. Panel **B** shows the marginal probability of hospital mortality by level of oxygen support. NC = nasal cannula (or other low flow oxygen); HF = high-flow oxygen; MV = mechanical ventilation (invasive or non-invasive). Panel **C** shows the marginal probability of hospital mortality by degree of parenchymal opacities on chest radiographs, determined by natural language processing of imaging reports. For Panels **B** and **C**: brackets indicate group-wise differences in logistic regression models. *** P < 0.001 ns = not significant

Among 765 patients, 201 met EHR-Berlin oxygenation criteria and 360 met imaging criteria within 24 h of admission (Fig. 2A). 121 patients met both criteria within 24 h, and relatively few patients qualified in each 24-hour period thereafter.

Next we quantified differences in outcomes by the level of oxygen support patients received at admission. Predicted probability of mortality ranged from 10% or less among patients on no or low-flow oxygen, to approximately 30% or greater among patients on high-flow and mechanical ventilation (Fig. 2B). Interestingly, the difference in odds of mortality among patients on high-flow oxygen compared to those on mechanical ventilation did not reach statistical significance (Fig. 2B; **eTable 4, Supplement**). Patients on high-flow oxygen did have significant differences in certain secondary outcomes, with greater ventilator-free days and higher odds of ordinal score ≤ 5 at day 14 (**eFigure 1, eTable 4, Supplement**).

When examining differences by imaging findings at admission, the predicted probability of mortality ranged from approximately 10% among patients without chest radiograph opacities, to 15% among patients with unilateral opacities, and > 20% among those with bilateral opacities (Fig. 2C; **eTable 5, Supplement**). As expected, odds of mortality among patients without opacities was significantly lower than patients with bilateral opacities. In contrast, odds of mortality among patients with unilateral opacities was not significantly different from patients with bilateral opacities, although they did experience better respiratory outcomes (**eFigure 1, eTable 5, Supplement**).

### Predictive validity of expanded ARDS definitions

**Fig. 3:**
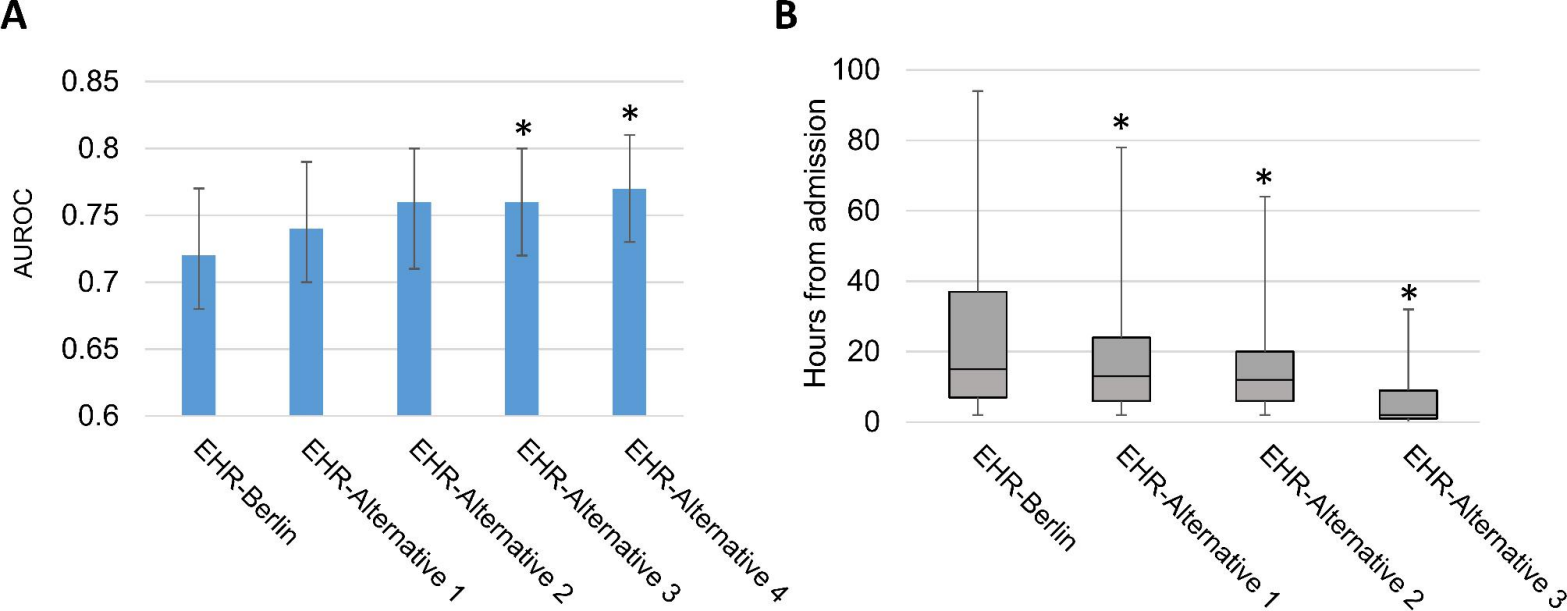
Comparison of Berlin and expanded EHR definitions. Panel **A** shows discrimination for hospital mortality by each computable ARDS definition, with blue bars indicating area under the receiver operating curve (AUROC), and error bars indicating 95% confidence interval. *P < 0.05 for Delong tests comparing to EHR-Berlin definition. Panel **B** shows boxplots of time (in hours) from hospital admission to meeting all ARDS criteria for each definition, among 171 patients who also met EHR-Berlin definition. Boxes indicate median (interquartile range) time, and whiskers indicate 10th and 90th percentile. EHR-Alternative 4 not plotted as it had the same imaging and oxygenation criteria as EHR-Alternative 3. *P < 0.001 for Wilcoxon signed rank tests comparing each alternative definition to EHR-Berlin definition

Overall, alternative ARDS definitions that successively expanded the oxygenation, imaging, and timing criteria had case prevalence ranging from 29% to 35%, and cases displayed similar baseline clinical features (**eTable 6, Supplement**). Associations between these ARDS definitions and outcomes were similar to those seen with the EHR-Berlin definition (**eTables 7–8, Supplement**). EHR-Alternative 1 (AUROC 0.74; 95% CI 0.70, 0.79; p = 0.14), which added patients who were hypoxemic while on high-flow oxygen, and EHR-Alternative 2 (AUROC 0.76, 95% CI 0.71, 0.80, p = 0.05), which then expanded imaging criteria to add patients with unilateral opacities, did not have significantly different discrimination for mortality compared to the EHR-Berlin definition (AUROC 0.72; 95% CI 0.68, 0.77) (Fig. 3A). EHR-Alternative 3 (AUROC 0.76; 95% CI 0.72, 0.80; p = 0.036) and EHR-Alternative 4 (AUROC 0.77, 95% CI 0.73, 0.81, p = 0.015), which removed imaging criteria altogether and then extended timing to 14 days, had significantly greater discrimination for mortality compared to EHR-Berlin definition. Last, we examined the extent to which definitions expanding oxygenation and imaging criteria enabled earlier identification of ARDS (Fig. 3B). The Berlin-EHR definition identified patients a median of 15 h from admission (interquartile range [IQR]: 7, 37 h), as compared with 13 h (IQR 6, 24) for EHR-Alternative 1 and 12 h (IQR 6, 20) for EHR-Alternative 2—differences that were statistically significant (P < 0.001). EHR-Alternative 3, which removed the chest imaging requirement, identified ARDS just 2 h (IQR 1, 9) from admission.

## Discussion

We provide evidence supporting the construct validity of an EHR-based ARDS definition among adults hospitalized with COVID-19, and then demonstrate how changes in the criteria of the definition affect predictive validity. The EHR-Berlin definition had high agreement with ARDS ascertainment by experts and was consistently linked to mortality and respiratory outcomes, thereby supporting both criterion and predictive validity. We then leveraged the tools we developed for this definition to investigate the validity of new ARDS definitions. Overall, we found liberalizing criteria served to not only classify a greater number of patients as ARDS, but also maintained consistent relationships with outcomes, prompted earlier diagnosis, and in some cases offered better discrimination for mortality. Taken together, the findings shed light on the implications for expanding ARDS definitions, while supporting the use of EHR-based approaches for identifying ARDS cases. Our findings also reinforce studies of acute respiratory failure that predate the pandemic, suggesting our work has relevance for not only for COVID-19 but also for traditional ARDS.

### The utility of computable definitions

It is critical to develop and assess the validity of pragmatic strategies for ARDS identification in real-world data [6, 7]. While other groups have also developed computable ARDS definition, only two prior studies also described a PPV over 90% [32–35]. Our Berlin-EHR definition is also unique from prior work by (1) incorporating SpO_2_ into oxygenation criteria[21, 36]; (2) using a novel NLP algorithm to determine bilateral opacities[17]; and (3) focusing on COVID-19. Our study also emphasizes the importance of using these complex data types over diagnosis codes or clinical documentation, though the latter are commonly used in computable case definitions for other conditions for their ease and portability across systems [25, 37, 38]. This is consistent with a small study of ICD-9 codes over 15 years ago, and multiple observational studies showing that clinicians under-recognize ARDS [2, 39–42]. Altogether, our computable EHR-Berlin definition may have such applications as diagnostic assistance in care settings, to facilitate delivery of evidence-based ARDS care, and larger-scale research, as manual ARDS ascertainment poses barriers to powering studies.

### Timing of ARDS classification

We found that over 70% of patients who eventually met criteria for the EHR-Berlin definition were identified within one day of admission. Similarly, the expanded definition that identified ARDS cases through 14 days of hospitalization (EHR-Alternative 4) found few additional patients compared to the definition limiting to 7 days (EHR-Alternative 3). Although others have reported delays between COVID-19 symptom onset and the development of respiratory failure, these findings suggest clinical progression largely occurs prior to hospitalization, and that patients quickly manifest imaging findings and hypoxemia after presentation.

Moreover, contemporary COVID-19 and ICU studies increasingly target enrollment to the earliest phases of illness, shortly after hospital or ICU admission [21, 28, 43]. Some alternative definitions could facilitate this goal, as they identified patients as ARDS significantly earlier than the Berlin definition. This ranged from two hours earlier with a definition that added patients on high-flow oxygen (EHR-Alternative 1), to 13 h earlier with definitions that removed imaging requirements (EHR-Alternative 3). While these differences seem modest, initiating treatment within two hours of critical illness has been strongly linked to improved outcomes in sepsis and ARDS [44–46].

### Expanding oxygenation criteria to add patients on high-flow oxygen

Many ARDS experts have proposed liberalizing the Berlin definition by including patients who are hypoxemic while on high-flow oxygen, because these patients are pathophysiologically similar, and high-flow is used commonly to prevent or delay mechanical ventilation [47, 48]. On the other hand, prior analyses have also suggested that classifying patients on high-flow oxygen as ARDS could be detrimental to interventional research, by enrolling a population with fewer disease-related outcomes like mortality and reducing statistical power [49]. Our work shows that even though patients on high-flow have somewhat lower mortality compared to those on mechanical ventilation, the differences were not significant. This helps explain why EHR-Alternative 1 still had substantial case mortality of 39% and maintained similar discrimination for hospital mortality compared the original Berlin-EHR definition. We posit that expanding study of respiratory failure beyond Berlin criteria may be appropriate for certain clinical scenarios and research questions, bringing attention to a larger set of patients that are still at high-risk for certain outcomes, and earlier in their illness course.

### Challenges with the imaging criteria of the Berlin definition

When determining the criterion validity of the Berlin-EHR definition, we determined the most common reason for disagreement was that the NLP determination of bilateral opacities did not match manual determinations made by our physicians. Although EHR-Berlin ARDS correctly classified 97% of patients, this mirrors prior work showing that chest imaging as a common source of discrepancy in ARDS diagnosis [50, 51]. While our computable definition does not address reliability of imaging interpretation, it has the distinct advantage of reducing the measurement burden and cost otherwise required for manual imaging review.

We also investigated the predictive validity of imaging criteria. First, we found that patients determined to have bilateral opacities by NLP, compared to those with unilateral opacities, did not have significantly worse mortality, although they did experience worse respiratory outcomes. Second, we found that a definition removing the imaging requirement altogether classified up to 51% more patients as ARDS compared to the Berlin definition, had higher discrimination for mortality, and similar correlations with other respiratory outcomes. We hypothesize that a factor contributing to this could be the limited sensitivity of chest radiographs for pulmonary edema, which may lead to under-diagnosis of ARDS [50–52]. Our findings are also consistent with prior work showing that patients who are ventilated and hypoxemic, even when they do not have bilateral opacities, are similar to Berlin ARDS in biologic features and mortality [53–55]. Together, the findings align with proposals to simplify radiographic criteria in COVID-19 ARDS, as a way to improve pragmatism and reproducibility of case identification [12].

### Limitations

Although we provide novel empiric data on the validity of several ARDS case definitions, it is important to recognize these properties may differ in other populations and settings, such as in traditional cohorts without EHR data, in other health systems, and in non-COVID-19 populations. Though our study included patients across 2 hospitals and 7 ICUs, it was in a single EHR and generalizability may be limited. Generalizability may be especially limited in low and middle income countries, where differences in ventilation practices and diagnostic resources could affect the validity of ARDS definitions [36]. Second, some of our analyses may have been limited by sample size. For example, relatively few patients were on high-flow oxygen compared to mechanical ventilation, which may have limited our statistical power to find differences in outcomes. Third, we chose to identify patients with bilateral opacities through NLP of imaging reports, which is more indirect than processing primary images. However, direct image analysis remains computationally expensive, and our approach is more practical for near-term use. Notwithstanding these limitations, our work demonstrates that pragmatic, automated approaches for identifying Berlin ARDS have high concordance with manual case identification, and highlights avenues for expanding Berlin ARDS criteria that capture a greater number of high-risk patients, earlier in their course.

### Limitations

## Conclusions

Computable ARDS definitions can support efficient, large-scale research and surveillance of high-risk patients, even when expanding beyond Berlin criteria.

### Electronic supplementary material

Below is the link to the electronic supplementary material.

Supplementary Material 1

## Data Availability

The datasets generated and analyzed during the current study are available from the corresponding author on reasonable request.

## List of abbreviations

ARDS: acute respiratory distress syndrome
EHR: electronic health records
PPV: positive predictive value
NPV: negative predictive value
PaO_2_/FIO_2_: ratio of arterial partial pressure oxygen to fraction of inspired oxygen
SpO_2_/FIO_2_: ratio of oxygen saturation to fraction of inspired oxygen
ICD-10: International Classification of Diseases Tenth Revision
COVID-19: coronavirus disease-2019
NLP: natural language processing
AUROC: area under the receiver operating curve
