# Supplementary material for: Evaluating construct validity of computable acute respiratory distress syndrome definitions in adults hospitalized with COVID-19: an electronic health records based approach
Source: BMC Pulm Med. 2023 Aug 9;23:292. doi: 10.1186/s12890-023-02560-y (PMC10413524; doi:10.1186/s12890-023-02560-y)
Supplement: Supplementary file 1 — Supplementary Material 1 [file 12890_2023_2560_MOESM1_ESM.pdf]

SUPPLEMENTAL CONTENT: Evaluating construct validity of acute respiratory distress syndrome definitions in adults hospitalized with COVID-19: an electronic health records based approach

AUTHORS: Neha A. Sathe, MD, MSc*[1]; Su Xian, MSc*[2]; F. Linzee Mabrey, MD[1]; David R. Crosslin, PhD[3]; Sean D. Mooney, PhD[2]; Eric D. Morrell, MD[1]; Kevin Lybarger, PhD[4]; Meliha Yetisgen, PhD[2]; Gail P. Jarvik, MD, PhD[5]; Pavan K. Bhatraju, MD, MSc[1]; Mark M. Wurfel, MD, PhD[1]

*authors contributed equally to this work

eFigure 1: Predicted respiratory outcomes by level of oxygen support and imaging findings. Panels A and B show predicted respiratory support- and ventilator-free days by level of oxygen support. NC = nasal cannula (or other low flow oxygen); HF = high-flow oxygen; MV = mechanical ventilation (invasive or non-invasive). Panel C shows the marginal probability of having an ordinal score ≤5 at day 14 by level of oxygen support. Panels D and E show predicted respiratory- and ventilator-free days by degree of parenchymal opacities on chest radiographs, determined by natural language processing of imaging reports. Panels F shows the marginal probability of having an ordinal score ≤5 at day 14 by degree of parenchymal opacities. For all panels: brackets indicate group-wise differences in linear or logistic regression models. *P < 0.05; **P < 0.01; ***P < 0.001; ns = not significant.

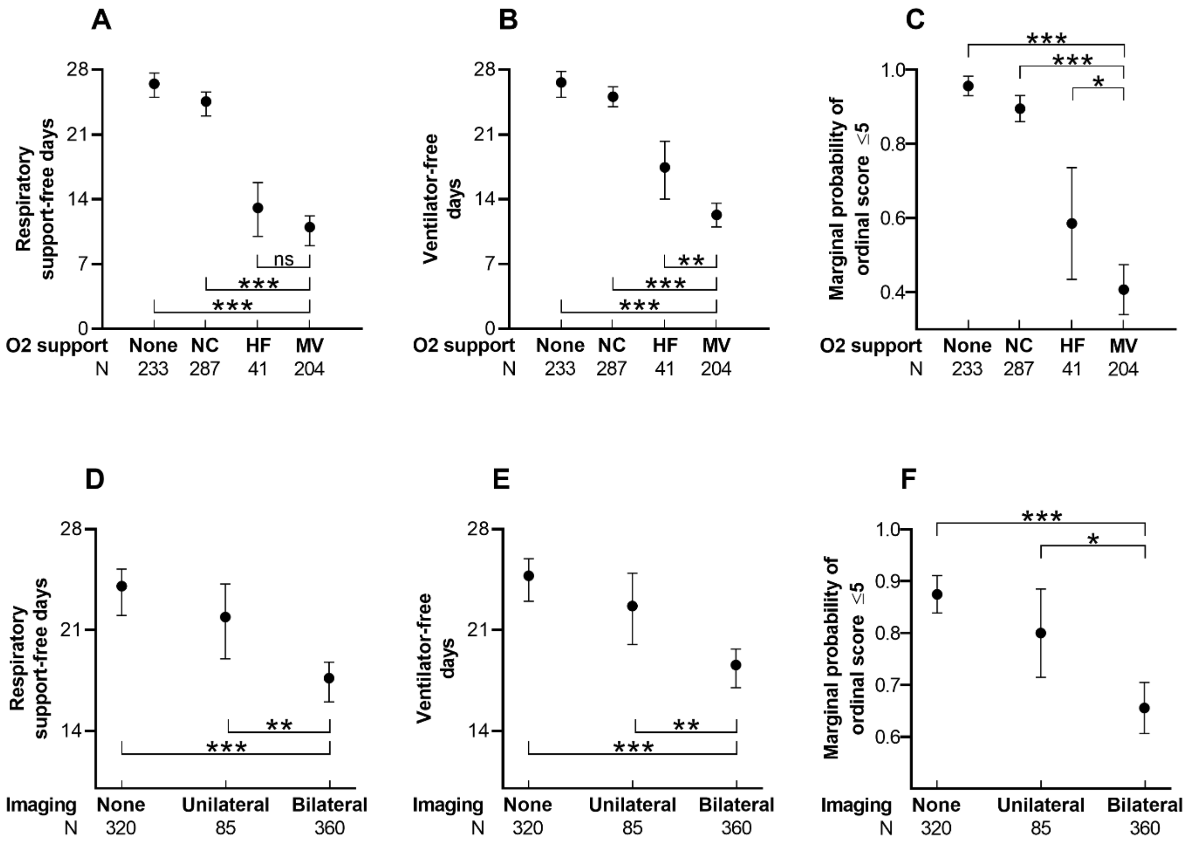

eTable 1: Computable ARDS case definitions

Definition	Oxygenation[a]	Timing[b]	Chest imaging[c]
**EHR-Berlin**	Qualifying PaO_2/FiO_2 or SpO_2/FiO_2 while on invasive mechanical ventilation or noninvasive mechanical ventilation	7 days	Bilateral opacities
**EHR-Alternative 1**	Qualifying PaO_2/FiO_2 or SpO_2/FiO_2 while on invasive mechanical ventilation, noninvasive mechanical ventilation, or high-flow oxygen	7 days	Bilateral opacities
**EHR-Alternative 2**	Qualifying PaO_2/FiO_2 or SpO_2/FiO_2 while on invasive mechanical ventilation, noninvasive mechanical ventilation, or high flow oxygen	7 days	Unilateral or bilateral opacities
**EHR-Alternative 3**	Qualifying PaO_2/FiO_2 or SpO_2/FiO_2 while on invasive mechanical ventilation, noninvasive mechanical ventilation, or high flow oxygen	7 days	No radiograph requirement
**EHR-Alternative 4**	Qualifying PaO_2/FiO_2 or SpO_2/FiO_2 while on invasive mechanical ventilation, noninvasive mechanical ventilation, or high flow oxygen	14 days	No radiograph requirement

Abbreviations: PaO_2/FiO_2 = ratio of arterial partial pressure oxygen to fraction of inspired oxygen. SpO_2/FiO_2 = ratio of oxygen saturation to fraction of inspired oxygen.

a. Has $PaO_2/FiO_2 \leq 300$ or if missing, a $SpO_2/FiO_2 \leq 315$ while on the specified oxygen support.
b. Meets oxygenation criterion within the specified time frame. Timing is from hospital admission.
c. Has a chest radiograph with the specified findings within the specified time frame. In addition we specified that the imaging criterion had to be met within 4 days of meeting oxygenation criterion. Chest radiograph classification determined by natural language processing of chest radiograph reports.

eTable 2. Agreement between EHR-Berlin definition and manual-Berlin reference standard

		**Manual-Berlin (reference standard)**		
		Yes	*No*	*Total*
**EHR-Berlin**	*Yes*	94	6	100
	No	7	68	75
	Total	101	74	175

Reference standard was ascertained among 175 COVID-19+ patients.

eTable 3. Reasons for discrepancies between EHR-Berlin definition and manual-Berlin reference standard

Type of misclassification	Description	Reason for discrepancy
False positive (n=6)	EHR-Berlin positive, manual-Berlin negative	5 patients had very brief and/or mild periods of hypoxemia that were not captured by initial manual chart review<br>1 patient did not meet chest imaging criteria on manual review
False negative (n=7)	EHR-Berlin negative, manual-Berlin positive	5 patients who did not meet chest imaging criterion by EHR-Berlin<br>2 patients did not meet oxygenation criterion by EHR-Berlin

eTable 4. Associations between level of oxygen support and clinical outcomes

	Mortality		Ordinal score ≤5 at day 14		Respiratory support-free days		Ventilator-free days	
	**OR (95% CI)**	**P**	**OR (95% CI)**	**P**	**Beta (95% CI)**	**P**	**Beta (95% CI)**	**P**
No supplemental oxygen	0.09 (0.04, 0.17)	<0.001	32.07 (16. 04, 64.11)	<0.001	15.48 (13.80,17.18)	<0.001	14.31 (12.57, 16.05)	<0.001
Low-flow oxygen	0.21 (0.13, 0.34)	<0.001	12.49 (7.80, 19.98)	<0.001	13.55 (11.94, 15.16)	<0.001	12.78 (11.12, 14.43)	<0.001
High-flow oxygen	0.78 (0.37, 1.6)	0.495	2.06 (1.04, 4.07)	0.038	2.08 (-0.93, 5.08)	0.175	5.14 (2.04, 8.23)	0.001
Mechanical ventilation	Reference		Reference		Reference		Reference	

Odds ratio (OR) for mortality and ordinal score associated with each level of oxygen calculated with logistic regression. Beta for respiratory support- and ventilator-free days associated with each level of oxygen calculated with linear regression.

eTable 5. Associations between imaging findings and clinical outcomes

	Mortality		Ordinal score ≤5 at day 14		Respiratory support-free days		Ventilator-free days	
	**OR (95% CI)**	**P**	**OR (95% CI)**	**P**	**β (95% CI)**	**P**	**β (95% CI)**	**P**
No parenchymal opacities	0.33 (0.21, 0.52)	<0.001	3.68 (2.47, 5.47)	< 0.001	6.39 (4.77)	<0.001	6.20 (4.59, 7.81)	<0.001
Unilateral opacities	0.62 (0.33, 1.18)	0.146	2.10 (1.18, 3.73)	0.011	4.25 (1.70, 6.80)	0.001	4.10 (1.57, 6.63)	0.002
Bilateral opacities	Reference		Reference		Reference		Reference	

Odds ratio (OR) for mortality and ordinal score associated with imaging findings calculated with logistic regression. Beta for respiratory support- and ventilator-free days associated with imaging findings calculated with linear regression.

eTable 6. Clinical features by expanded ARDS definitions

	EHR-Alternative 1			EHR-Alternative 2			EHR-Alternative 3			EHR-Alternative 4		
	ARDS-	ARDS+	P	ARDS-	ARDS+	P	ARDS-	ARDS+	P	ARDS-	ARDS+	P
	N=546	N=219		N=529	N=236		N=506	N=259		N=498	N=267	
**Demographics**												
Age, years	56 (19)	59 (15)	0.060	56 (19)	58 (15)	0.17	57 (19)	57 (16)	0.70	56 (19)	58 (16)	0.36
Male sex, N (%)	315 (58%)	156 (71%)	<0.001	303 (57%)	168 (71%)	<0.001	287 (57%)	184 (71%)	<0.001	281 (56%)	190 (71%)	<0.001
Race			<0.001			0.003			0.002			0.004
White	342 (63%)	136 (62%)		333 (63%)	145 (61%)		320 (63%)	158 (61%)		315 (63%)	163 (61%)	
Black/African American	95 (17%)	21 (10%)		91 (17%)	25 (11%)		87 (17%)	29 (11%)		85 (17%)	31 (12%)	
Asian	69 (13%)	30 (14%)		67 (13%)	32 (14%)		64 (13%)	35 (14%)		63 (13%)	36 (13%)	
American Indian or Alaskan Native	17 (3%)	7 (3%)		15 (3%)	9 (4%)		15 (3%)	9 (3%)		15 (3%)	9 (3%)	
Native Hawaiian or Pacific Islander	9 (2%)	5 (2%)		9 (2%)	5 (2%)		8 (2%)	6 (2%)		8 (2%)	6 (2%)	
Unknown	14 (3%)	20 (9%)		14 (3%)	20 (8%)		12 (2%)	22 (8%)		12 (2%)	22 (8%)	
Ethnicity			<0.001			0.001			0.002			0.005
Not Hispanic	405 (74%)	134 (61%)		392 (74%)	147 (62%)		376 (74%)	163 (63%)		369 (74%)	170 (64%)	
Hispanic	109 (20%)	67 (31%)		105 (20%)	71 (30%)		101 (20%)	75 (29%)		100 (20%)	76 (28%)	
Unknown	32 (6%)	18 (8%)		32 (6%)	18 (8%)		29 (6%)	21 (8%)		29 (6%)	21 (8%)	
**Chronic comorbidities**												
Diabetes, N (%)	211 (39%)	103 (47%)	0.033	204 (39%)	110 (47%)	0.037	197 (39%)	117 (45%)	0.097	192 (39%)	122 (46%)	0.056
Chronic renal disease, N (%)	147 (27%)	55 (25%)	0.61	140 (26%)	62 (26%)	0.96	137 (27%)	65 (25%)	0.56	135 (27%)	67 (25%)	0.55
Chronic heart failure, N (%)	142 (26%)	57 (26%)	>0.99	137 (26%)	62 (26%)	0.91	132 (26%)	67 (26%)	0.95	127 (26%)	72 (27%)	0.66
Chronic Pulmonary Disease, N (%)	123 (23%)	51 (23%)	0.82	117 (22%)	57 (24%)	0.54	116 (23%)	58 (22%)	0.87	112 (22%)	62 (23%)	0.82
**Illness severity at admission**												
Intensive care unit, N (%)	103 (19%)	152 (70%)	<0.001	92 (17%)	163 (70%)	<0.001	76 (15%)	179 (70%)	<0.001	76 (15%)	179 (68%)	<0.001
Invasive mechanical ventilation, N (%)	31 (6%)	113 (52%)	<0.001	22 (4%)	122 (52%)	<0.001	10 (2%)	134 (52%)	<0.001	10 (2%)	134 (50%)	<0.001
Noninvasive mechanical ventilation, N (%)	5 (1%)	16 (7%)	<0.001	5 (1%)	16 (7%)	<0.001	4 (1%)	17 (7%)	<0.001	4 (1%)	17 (6%)	<0.001
High-flow oxygen, N (%)	4 (1%)	38 (17%)	<0.001	2 (0%)	40 (17%)	<0.001	0 (0%)	42 (16%)	<0.001	0 (0%)	42 (16%)	<0.001
**Resource utilization outcomes**												
Duration of invasive mechanical ventilation, days, median (IQR)	0 (0-0)	7 (1-17)	<0.001	0 (0-0)	6 (1-17)	<0.001	0 (0-0)	6 (1-16)	<0.001	0 (0-0)	5 (1-15)	<0.001
ICU length of stay, days, median (IQR)	0 (0-1)	10 (4-20)	<0.001	0 (0-0)	9 (4-19)	<0.001	0 (0-0)	9 (3-19)	<0.001	0 (0-0)	8 (3-18)	<0.001
Hospital length of stay, days, median (IQR)	6 (3-12)	17 (10-28)	<0.001	6 (3-11)	17 (10-28)	<0.001	6 (3-11)	17 (9-28)	<0.001	6 (3-11)	17 (9-28)	<0.001

eTable 7. Clinical outcomes by expanded definitions

	EHR-Alternative 1			EHR-Alternative 2			EHR-Alternative 3			EHR-Alternative 4		
	**ARDS-**	**ARDS+**	**P**	**ARDS-**	**ARDS+**	**P**	**ARDS-**	**ARDS+**	**P**	**ARDS-**	**ARDS+**	**P**
	N=546	N=219		N=529	N=236		N=506	N=259		N=498	N=267	
Mortality	37 (7%)	85 (39%)	<0.001	32 (6%)	90 (38%)	<0.001	27 (5%)	95 (37%)	<0.001	24 (5%)	98 (37%)	<0.001
Respiratory support free days	28 (28-28)	3 (0-21)	<0.001	28 (28-28)	4 (0-22)	<0.001	28 (28-28)	6 (0-22)	<0.001	28 (28-28)	7 (0-23)	<0.001
Ventilator free days	28 (28-28)	5 (0-26)	<0.001	28 (28-28)	10 (0-26)	<0.001	28 (28-28)	10 (0-27)	<0.001	28 (28-28)	11 (0-27)	<0.001
Ordinal score ≤ 5 at day 14	500 (92%)	84 (38%)	<0.001	488 (92%)	96 (41%)	<0.001	478 (94%)	106 (41%)	<0.001	474 (95%)	110 (41%)	<0.001

ARDS- and ARDS+ columns indicate N (%) for categorical outcomes (mortality and ordinal score) and median (interquartile range) of continuous outcomes (respiratory support- and ventilator-free days) by patients who were not and who were classified as ARDS, respectively. P values are for Chi square tests for categorical outcomes and Wilcoxon rank sum tests for continuous outcomes.

eTable 8: Association between expanded definitions and outcomes

Expanded definitions	Hospital mortality		Respiratory support-free days		Ventilator-free days		Ordinal score ≤ 5 at day 14	
	r	**OR (95% CI)**	***r***	**β (95% CI)**	***r***	**β (95% CI)**	***r***	**OR (95% CI)**
EHR-Alternative 1	0.40	8.73 (5.67, 13.42)	-0.63	-15.62 (-16.98, -14.26)	-0.57	-13.89 (-15.32, 12.46)	-0.57	0.06 (0.04, 0.09)
EHR-Alternative 2	0.40	9.57 (6.14, 14.92)	-0.64	-15.42 (-16.75, -14.10)	-0.58	-13.77 (-15.16, -12.38)	-0.56	0.06 (0.04, 0.09)
EHR-Alternative 3	0.41	10.28 (6.47, 16.32)	-0.66	-15.58 (-16.84, -14.31)	-0.58	-13.63, (-14.98, -12.29)	-0.60	0.04 (0.03, 0.06)
EHR-Alternative 4	0.42	11.45 (7.09), 18.80)	-0.67	-15.64 (-16.88, -14.40)	-0.59	-13.66 (-14.99, -12.32)	-0.61	0.04 (0.02, 0.06)

r indicates Pearson correlation coefficient between ARDS classification and each outcome. Odds ratio (OR) calculated with logistic regression for categorical outcomes (mortality and ordinal score), beta (β) calculated with linear regression for continuous outcomes (respiratory support- and ventilator-free days).
